# DOMINÓ Registry: study protocol on mineral and bone disease (DOença MINeral e Óssea) of chronic kidney disease in pediatrics in Brazil

**DOI:** 10.1590/2175-8239-JBN-2024-0054en

**Published:** 2024-12-20

**Authors:** Emília Maria Dantas Soeiro, Maria Goretti Moreira Guimarães Penido, Lucimary de Castro Sylvestre, Maria Cristina Andrade, Suzana Aparecida Greggi de Alcantara, Ivan Coelho Machado, Leonardo Gonçalves Bedram, Ana Lucia Santos Abreu

**Affiliations:** 1 Universidade Federal de Pernambuco, Recife, PE, Brazil.; 2 Faculdade Pernambucana de Saúde, Recife, PE, Brazil.; 3 Instituto de Medicina Integral Professor Fernando Figueira, Recife, PE, Brazil.; 4 Santa Casa de Belo Horizonte, Centro de Nefrologia, Unidade de Nefrologia Pediátrica, Belo Horizonte, MG, Brazil.; 5 Universidade Federal de Minas Gerais, Faculdade de Medicina, Unidade de Nefrologia Pediátrica, Hospital das Clínicas, Belo Horizonte, MG, Brazil.; 6 Hospital Pequeno Príncipe, Unidade de Nefrologia Pediátrica, Curitiba, PR, Brazil.; 7 Pontifícia Universidade Católica do Paraná, Curitiba, PR, Brazil.; 8 Universidade Federal de São Paulo, Escola Paulista de Medicina, São Paulo, SP, Brazil.; 9 Universidade de São Paulo, Faculdade de Medicina de Ribeirão Preto, Hospital das Clínicas, Ribeirão Preto, SP, Brazil.

**Keywords:** Chronic Kidney Disease-Mineral and Bone Disorder, Renal Insufficiency, Chronic, Treatment, Diagnosis, Treatment Outcome, Registries

## Abstract

**Introduction:**

Pediatric patients with chronic kidney disease (CKD) develop mineral and bone disorders (MBD). We do not have Brazilian data that evaluate these outcomes, which can be obtained through epidemiological records.

**Objective:**

To present the DOMINÓ study, which aims to describe CKD-MBD characteristics in Brazilian pediatric patients.

**Methods:**

Retrospective and prospective, multicenter, observational cohort. The retrospective study will analyze data from prevalent patients in 2024, and the prospective study will analyze data from 2025 onwards. Demographic, clinical, laboratory, imaging, and bone biopsy data will be collected from pediatric patients with CKD-MBD < 18 years old with CKD stage 3-5D and kidney transplant recipients. The Ethics Committees of the participating centers approved the study.

**Discussion/conclusion:**

The DOMINÓ study will provide information on the incidence, prevalence, morbidity, treatment results, and mortality of this pediatric disease in Brazil. Future analyses will allow us to identify predictors of response to treatment and improve the care for these patients.

## Introduction

Mineral and bone metabolism disorder associated with chronic kidney disease (CKD-MBD) is part of a broad clinical spectrum involving bone changes, alterations in mineral metabolism parameters, and extraskeletal calcifications^1,2^. In the pediatric population, these changes manifest in the early stages of CKD, and worsen as renal function declines^3^. In the growing skeleton, CKD-MBD affects bone modeling and remodeling, compromising mineral accumulation and deposition in bones^3,4^. These alterations manifest as growth restriction, bone deformities, epiphysiolysis, and fractures, affecting the quality of life of these patients^3^. At the same time, the formation of vascular calcifications and the progression of cardiovascular disease contribute to the death of these children^5^.

Data from international literature show that the incidence of fractures in pediatric patients with CKD is a threefold higher than in their healthy peers, and this risk persists into adulthood^3^. Even after kidney transplantation, the risk remains six times higher than in the population from the same age group^3^. In addition, half of children with CKD do not reach the genetic height target^3^. Children and young adults with CKD develop early vascular calcification, even when MBD improves^5^. It is noteworthy that cardiovascular disease accounts for up to 30% of deaths in children undergoing dialysis^6,7^.

Regarding the diagnosis of CKD-MBD in the pediatric population, serum parathyroid hormone (PTH) levels increase at an early stage. Along with alkaline phosphatase (ALP), they are considered markers of bone remodeling. Despite the great serum variation of these markers in pediatric studies, high-remodeling disease is characterized by elevated serum PTH and ALP, while in low-remodeling disease serum PTH and ALP are lower^8^. Novel, more specific markers of bone formation and resorption have been studied to establish correlations with bone histology; however, only PTH and ALP have proven useful in pediatric clinical practice^9^. As for the evaluation of imaging tests in pediatrics, there are no current recommendations for performing densitometry, high-resolution tomography, or MRI. Similarly, lateral abdominal radiography to assess vascular calcifications has not yet been well established for this age group^1,10,11^. Other tests, such as pulse wave variation, carotid Doppler ultrasound and computed tomography calcium scoring, are indicated in specific cases and in research setting^10^. In clinical practice, plain radiography is usually used to assess bone age, fractures, signs of rickets, late manifestations of hyperparathyroidism, and metastatic calcifications^10^. Histological analysis of bone tissue obtained through biopsy is the gold standard for diagnosing CKD-MBD. Bone biopsy is recommended in children with CKD in cases of severe bone deformity or pain, atraumatic fractures, persistent hypercalcemia or hypophosphatemia, despite optimized treatment^10^.

Despite the severity of CKD-MBD in the pediatric age group, the diagnosis is clearly complex. International registries studying children with CKD-MBD^12^, as well as national studies on adults^13,14^, stand out as valuable tools for understanding this issue. In Brazil, there is no multicenter study on pediatric CKD-MBD. Following the example of the contributions made by these registries, the need for a Brazilian registry on pediatric CKD-MBD is evident.

In its initial phase, the DOMINÓ project aims at describing, in general terms, the epidemiological, clinical, laboratory, radiological, and bone biopsy characteristics of CKD-MBD in Brazilian pediatric patients. The specific objectives include: (1) identifying the prevalence of CKD-MBD by means of biochemical profiles, (2) analyzing the association between CKD-MBD and clinical data, morbidity (fractures, growth deficit, deformities, and bone pain), and mortality, (3) proposing diagnostic and treatment guidelines for pediatric CKD-MBD.

The prospective observational study will analyze outcomes such as growth, cardiovascular morbidity, fractures, response to treatment, and mortality based on six-monthly data collection and annual assessment over a three-year period, which may extend.

## Methods

### Study Design and Population

The DOMINÓ Registry will be a retrospective and prospective, multicenter, observational cohort study. The initial phase will analyze data from prevalent patients in 2024, while the prospective study will analyze data from 2025 onwards. Pediatric patients with CKD stages 3 to 5-D, and kidney transplant recipients, aged up to 17 years, 11 months, and 29 days, from the respective centers will be eligible.

The sample will be one of convenience, as all children and adolescents from the participating centers who meet the inclusion criteria will be invited to attend. A sample of 700 patients is estimated. Collection will be carried out by researchers from the respective centers, using data from medical records.

Patients whose parents/legal guardians agree to participate and sign the informed consent form will be included, and for participants over the age of 10, an informed assent form must also be duly signed.

### Analysis Variables

*Demographic variables*: Center name, patient number, patient initials, date of birth, sex, race/skin color, CKD etiology, date of follow-up initiation, type of CKD treatment, date of 1st dialysis initiation, dialysis modality, date of dialysis modality change, transplant date, date of current evaluation. These data are detailed in Table 1.

**Table 1:** Demographic data

Demographic data
Center name Patient number Patient initials Date of birth Sex (1. Female; 2. Male) Race/skin color (1. White; 2. Non-white) CKD etiology (1. Uropathy; 2. Glomerulopathy; 3. Hereditary diseases; 4. Cystic diseases; 5. Others) Date of CKD diagnosis/Follow-up initiation Type of CKD treatment (1. Conservative; 2. Dialysis; 3. Transplant) Date of first dialysis initiation Dialysis modality (1. Peritoneal; 2. Hemodialysis; 3. Hemodiafiltration) Date of dialysis modality change (if any) Transplant date Date of current evaluation

*Anthropometric and clinical data on bone involvement*: Weight, height, and body mass index (BMI) according to the World Health Organization classifications. Data such as: bone pain, fractures, fracture site, bone deformity, limited ambulation due to bone impairment, brown tumor, and other signs and symptoms will be categorized (yes/no) Table 2.

**Table 2:** Anthropometric, clinical, and treatment data

Anthropometric data
Weight Z-score weight/age Height (cm) Z-score height/age Height classification (1. short stature; 2. adequate stature; 3. tall stature) Body Mass Index (BMI) BMI Z-score BMI classification (1. underweight; 2. normal weight; 3. overweight/obese)
Clinical data on bone involvement
Bone pain (1. Yes; 2. No) Fractures (1. Yes; 2. No) Bone deformity (1. Yes; 2. No) Fracture site (1. Lower limb; 2. Upper limb; 3. Spine; 4. Hip; 5. Others) Signs of osteopenia (1. Yes; 2. No) Limited ambulation due to bone impairment (1. Yes; 2. No) Brown tumor (1. Yes; 2. No) Other symptoms (1. Yes; 2. No)
CKD-MBD treatment data
Type of phosphate binder used (1. No use; 2. Calcium carbonate; 3. Sevelamer; 4. Others) Dose of calcium carbonate binder (dose in mg/kg/day) Dose of calcium carbonate supplement (dose in mg/kg/day) Dose of sevelamer (dose in mg/kg/day) Calcitriol use (1. Yes; 2. No) Calcitriol dose (mcg/kg/week) Paricalcitol use (1. Yes; 2. No) Paricalcitol dose (mg/kg/day) Cinacalcet use (1. Yes; 2. No) Cinacalcet dose (mg/kg/day) Sodium bicarbonate (1. Yes; 2. No) Sodium bicarbonate dose (mEq/kg/day) Corticosteroid use (1. Yes; 2. No) Calcium concentration in the dialysate (mmol/L) (1. 1.25; 2. 1.5; 3. 1.75; 4. Other)

*Data related to CKD-MBD treatment*: Type and dose of phosphate binder, vitamin D or analogues (cholecalciferol, calcitriol, paricalcitol, others), calcimimetic, sodium bicarbonate, corticosteroid. Calcium concentration in the dialysate. The types of medication will be analyzed as categorical variables and the dose as a continuous variable (dose per kilo of body weight) Table 2.

*Laboratory tests*: Serum calcium (mg/dL), serum phosphorus (mg/dL), alkaline phosphatase (IU/L), gamma-glutamyl transferase (γGT U/L), 25-hydroxyvitamin D (ng/mL), 1,25-dihydroxyvitamin D (ng/mL), iPTH (pg/mL), serum bicarbonate (mEq/L). Normality values will be defined and assessed according to current protocols and guidelines^11^. Since the normal ranges for alkaline phosphatase and iPTH assays may not be the same between centers, data for these markers will be standardized against the upper limit of normal (ULN). These data are detailed in Table 3.

**Table 3:** Data from laboratory and imaging tests

Laboratory data
eGFR (conservative treatment and transplanted patients) (Schwartz formula) Creatinine (mg/dL) Creatinine dosage (1. Jaffé; 2. enzymatic) Urea (mg/dL) Calcium (mg/dL) Phosphorus (mg/dL) Bicarbonate (mEq/L) ALP (UI/L) ALP RV (minimum) ALP RV (maximum) ALP (xULN) PTH (pg/mL) PTH RV (minimum) PTH RV (maximum) PTH (xULN) 25-hydroxyvitamin D (ng/mL) 1,25-dihydroxyvitamin D (ng/mL) Gamma-glutamyl transferase (U/L)
Plain radiography
Bone age in months (0. None; 1. Normal; 2. Delayed; 3. Advanced) Hand and wrist (0/1/2/3) Tibia (0/1/2/3) Pelvis (0/1/2/3) Skull (0/1/2/3) Lateral abdomen (0/1/2/3) (0. Not performed; 1. Normal; 2. Signs of osteopenia; 3. Fracture) Soft tissue calcification (1. Yes; 2. No) Fracture at another site (1. Yes; 2. No)
Bone densitometry
Bone densitometry performed (1. Yes; 2. No) BMD (L1-L4) (g/cm²) BMC (L1-L4) (g) Area (cm^2^) % reference database (%) BMC/bone size (g/cm^3^)
Echocardiography
Echocardiography (1. Yes; 2. No) LV mass (g) Mass index (g/m^2,7^) Diastolic dysfunction (1. Yes; 2. No) Ejection fraction (%) Valve calcification (1. Yes; 2. No)

Abbreviations – ALP: alkaline phosphatase; PTH: parathyroid hormone; RV: reference value; xULN: number of times the upper limit of normal; BMC: bone mineral content; BMD: bone mineral density.

*Imaging tests*: X-rays to assess bone age, osteopenia, deformities and fractures will be analyzed by the radiologist at each institution, and the data will be categorized (yes/no). Bone densitometry will follow recent recommendations^9,15^. Echocardiography will assess myocardial dysfunction and valve calcification (Table 3).

*Bone biopsy data*: When performed based on clinical indication, we will assess the histological diagnosis using TMV classification [Turnover (remodeling), Mineralization, and Volume]^1^, in addition to other histomorphometry parameters, detailed in the supplementary table.

### Data Processing and Analysis

The variables will be collected in a structured form using Excel^®^. Each center will have its own spreadsheet, which should undergo double-checking. We are in the process of approving a registry for data to be entered electronically, securely, and privately at the Brazilian Society of Nephrology, where it will be entered by authorized, registered, and duly authenticated physicians.

Continuous and semi-continuous data will be analyzed using the Shapiro-Wilk test to determine normality. Mean and standard deviation, or median and interquartile range, will be expressed as appropriate. Comparisons and correlations will be conducted using parametric and non-parametric tests accordingly. Categorical data will be analyzed using the chi-square test with significance. To avoid a type I error, we set α level at ≤ 0.05 for the entire study.

### Ethical Aspects

This study was designed following the Regulatory Guidelines and Norms for Research Involving Human Subjects set forth by the National Health Council, and proposed by Resolutions 466/12 and 510/16, aiming to preserve the four principles of bioethics: autonomy, nonmaleficence, beneficence, and justice. Furthermore, it meets the requirements of the Declaration of Helsinki for research on human beings. We currently have five centers approved by their respective Ethics Committees, with plans to include additional centers in Brazil, subject to approval by the corresponding research ethics committee.

### Discussion

Considering the high morbidity and mortality of pediatric CKD-MBD, diagnosing this entity is extremely important. Assessment of bone health and cardiovascular involvement are key elements in the care of these children and adolescents.

As previously mentioned, studies on pediatric CKD-MBD in our country are scarce. Analyzing data from the DOMINÓ registry may reveal predictors of treatment response for CKD-MBD.

The challenges of this study may include the number of tables to be completed in and the lack of interest from parents/caregivers in signing the informed consent form. Despite the obstacles, it is important to know the epidemiological, clinical and therapeutic data on this disease to improve morbidity, mortality and the quality of life for these patients. The DOMINÓ study will serve as a comprehensive data registry for pediatric CKD-MBD in Brazil, enabling future research into this major issue of CKD among children and adolescents.

## Supplementary material

The following online material is available for this article:

Table S1 - Bone biopsy data.
