# Supplementary material for: DOMINÓ Registry: study protocol on mineral and bone disease (DOença MINeral e Óssea) of chronic kidney disease in pediatrics in Brazil
Source: J Bras Nefrol. 2024 Dec 20;47(1):e20240054. doi: 10.1590/2175-8239-JBN-2024-0054en (PMC11706022; doi:10.1590/2175-8239-JBN-2024-0054en)
Supplement: Supplementary file 1 [file 2175-8239-jbn-47-1-e20240054-suppl.pdf]

Braz. J. Nephrol.
https://doi.org/10.1590/2175-8239-JBN-2024-0054en

Supplementary Material to "DOMINÓ Registry: Study protocol on Mineral and Bone Disease (DOença MINeral e Óssea) of Chronic Kidney Disease in Pediatrics in Brazil"

Table S1 - Bone biopsy data.

**Bone Biopsy data**		
Bone biopsy (1-yes, 2-no)		
Bone biopsy (1-normal, 2- osteitis fibrosa, 3- osteomalacia, 4-adynamic bone disease, 5- mixed disease)		
**TMV Classification**		
Turnover (1-low 2-normal, 3-high)	Mineralization (1- normal, 2- abnormal)	Volume (1-low, 2- normal, 3- high)
**Metal deposition**		
Iron surface (Fe.S/BS) %	Aluminum surface (Al.S/BS) %	
**Structural parameters**		
Bone volume BV/TV(%)	Trabecular thickness Tb.Th (µm)	
Trabecular number Tb.N (/mm ou mm-1)	Trabecular separation Tb.Sp (µm)	
**Bone formation parameters**		
Osteoid thickness O.Th (µm)	Osteoid surface OS/BS (%)	
Osteoid volume OV/BV (%)	Osteoblast surface Ob.S/BS (%)	
**Bone resorption parameters**		
Eroded surface ES/BS (%)	Osteoclast surface Oc.S/BS (%)	
Fibrosis volume Fb.V/TV (%)		
**Mineralization parameters**		
Mineralizing surface MS/BS (%)	Bone formation rate BFR/BS (µm3/µm2/dia)	
Mineral apposition rate MAR (µm/dia)	Adjusted apposition rate AJ.AR (µm/dia)	
Mineralization lag time Mlt (dias)		
