# Supplementary material for: DOMINÓ Registry: study protocol on mineral and bone disease (DOença MINeral e Óssea) of chronic kidney disease in pediatrics in Brazil
Source: J Bras Nefrol. 2024 Dec 20;47(1):e20240054. doi: 10.1590/2175-8239-JBN-2024-0054en (PMC11706022; doi:10.1590/2175-8239-JBN-2024-0054en)
Supplement: Supplementary file 2 [file 2175-8239-jbn-47-1-e20240054-pt-suppl.pdf]

Braz. J. Nephrol. (J. Bras. Nefrol.)
https://doi.org/10.1590/2175-8239-JBN-2024-0054pt

Material Suplementar para "Registro DOMINÓ: Protocolo do estudo da DOença MINeral e Óssea da Doença Renal Crônica em pediatria no Brasil"

Tabela S1 - Dados de biópsia óssea.

**Dados de biópsia óssea**		
Biopsia óssea (1. Sim; 2. Não)		
Biopsia óssea (1. Normal; 2. Osteíte fibrosa; 3. Osteomalácia; 4. Doença adinâmica; 5. Doença mista)		
**Classificação TMV**		
Remodelação<br>(1. Baixa; 2. Normal; 3. Alta)	Mineralização<br>(1. Normal; 2. Anormal)	Volume<br>(1. Baixo; 2. Normal; 3. Aumentado)
**Depósito de metais**		
Superfície recoberta por ferro (Fe.S/BS) %	Superfície recoberta por alumínio (Al.S/BS) %	
**Parâmetros estruturais**		
Volume ósseo BV/TV(%)	Espessura trabecular Tb.Th (μm)	
Número de trabéculas Tb.N (/mm ou mm. 1)	Separação trabecular Tb.Sp (μm)	
**Parâmetros estáticos de formação óssea**		
Espessura osteoide O.Th (μm)	Superfície osteoide OS/BS (%)	
Volume osteoide OV/BV (%)	Superfície osteoblástica Ob.S/BS (%)	
**Parâmetros estáticos de reabsorção**		
Superfície de reabsorção ES/BS (%)	Superfície osteoclástica Oc.S/BS (%)	
Volume de fibrose Fb.V/TV (%)		
**Parâmetros de mineralização**		
Superfície de mineralizante MS/BS (%)	Taxa de formação óssea BFR/BS (μm3/μm2/dia)	
Taxa de aposição mineral MAR (μm/dia)	Taxa de formação óssea corrigida AJ.AR (μm/dia)	
Intervalo de tempo para a mineralização Mlt (dias)		
