# Supplementary material for: DOMINÓ Registry: study protocol on mineral and bone disease (DOença MINeral e Óssea) of chronic kidney disease in pediatrics in Brazil
Source: J Bras Nefrol. 2024 Dec 20;47(1):e20240054. doi: 10.1590/2175-8239-JBN-2024-0054en (PMC11706022; doi:10.1590/2175-8239-JBN-2024-0054en)
Supplement: Translation

# Translation: Registro DOMINÓ: protocolo do estudo da DOença MINeral e Óssea da doença renal crônica em pediatria no Brasil

**DOI:** 10.1590/2175-8239-JBN-2024-0054pt

**Authors:** Emília Maria Dantas Soeiro, Maria Goretti Moreira Guimarães Penido, Lucimary de Castro Sylvestre, Maria Cristina Andrade, Suzana Aparecida Greggi de Alcantara, Ivan Coelho Machado, Leonardo Gonçalves Bedram, Ana Lucia Santos Abreu

**Affiliations:** 1 Universidade Federal de Pernambuco, Recife, PE, Brasil.; 2 Faculdade Pernambucana de Saúde, Recife, PE, Brasil.; 3 Instituto de Medicina Integral Professor Fernando Figueira, Recife, PE, Brasil.; 4 Santa Casa de Belo Horizonte, Centro de Nefrologia, Unidade de Nefrologia Pediátrica, Belo Horizonte, MG, Brasil.; 5 Universidade Federal de Minas Gerais, Faculdade de Medicina, Unidade de Nefrologia Pediátrica, Hospital das Clínicas, Belo Horizonte, MG, Brasil.; 6 Hospital Pequeno Príncipe, Unidade de Nefrologia Pediátrica, Curitiba, PR, Brasil.; 7 Pontifícia Universidade Católica do Paraná, Curitiba, PR, Brasil.; 8 Universidade Federal de São Paulo, Escola Paulista de Medicina, São Paulo, SP, Brasil.; 9 Universidade de São Paulo, Faculdade de Medicina de Ribeirão Preto, Hospital das Clínicas, Ribeirão Preto, SP, Brasil.

**Keywords:** Distúrbio Mineral e Ósseo na Doença Renal Crônica, Insuficiência Renal Crônica, Tratamento, Diagnóstico, Resultado do Tratamento, Sistema de Registros

## Abstract

**Introdução:**

Pacientes pediátricos com doença renal crônica (DRC) evoluem com distúrbios minerais e ósseos (DMO). Não dispomos de dados brasileiros que avaliem esses desfechos; que podem ser obtidos por meio de registros epidemiológicos.

**Objetivo:**

Apresentar o projeto DOMINÓ, cuja proposta é descrever as características da DMO-DRC em pacientes pediátricos brasileiros.

**Métodos:**

Coorte retrospectiva e prospectiva, multicêntrica, observacional. O estudo retrospectivo analisará os dados dos pacientes prevalentes em 2024 e o prospectivo, os dados a partir de 2025. Serão coletados dados demográficos, clínicos, laboratoriais, de imagem e da biópsia óssea de pacientes pediátricos com DMO-DRC < 18 anos com DRC estágios 3-5D e dos transplantados renais. Os Comitês de Ética dos centros participantes aprovaram o estudo.

**Discussão/conclusão:**

O estudo DOMINÓ permitirá conhecer a incidência, prevalência, morbidade, resultados dos tratamentos e mortalidade dessa doença pediátrica no Brasil. Análises futuras permitirão identificar preditores de resposta ao tratamento e melhorar os cuidados com esses pacientes.

## Introdução

O distúrbio do metabolismo mineral e ósseo associado à doença renal crônica (DMO-DRC) faz parte de um espectro clínico amplo, envolvendo alterações ósseas, alterações nos parâmetros do metabolismo mineral e calcificações extraesqueléticas^1,2^. Na população pediátrica, essas mudanças se manifestam nos estágios iniciais da DRC e pioram à medida que a função renal se agrava^3^. No esqueleto em crescimento, a DMO-DRC afeta a modelação e a remodelação ósseas, comprometendo o acúmulo e a deposição mineral nos ossos^3,4^. Essas alterações se manifestam como restrição de crescimento, deformidades ósseas, epifisiólise e fraturas, afetando a qualidade de vida desses pacientes^3^. Paralelamente, a formação de calcificações vasculares e a evolução da doença cardiovascular contribuem para o óbito dessas crianças^5^.

Dados da literatura internacional mostram que a incidência de fraturas em pacientes pediátricos com DRC é três vezes maior do que em seus pares saudáveis, e esse risco se mantém na vida adulta^3^. Mesmo após o transplante renal, o risco permanece seis vezes maior que na população na mesma faixa etária^3^. Além disso, metade das crianças com DRC não atinge o alvo de estatura genético^3^. Crianças e adultos jovens com DRC desenvolvem precocemente calcificação vascular, mesmo quando há melhora na DMO^5^. Ressalta-se que a doença cardiovascular é responsável por até 30% das mortes em crianças em diálise^6,7^.

Em relação ao diagnóstico da DMO-DRC na população pediátrica, os níveis séricos de paratormônio (PTH) se elevam precocemente e junto à fosfatase alcalina (FA) são considerados marcadores de remodelação óssea. Apesar da grande variação sérica desses marcadores nos estudos pediátricos, a doença de alta remodelação se caracteriza por PTH e FA séricos elevados, enquanto que na doença de baixa remodelação o PTH e a FA séricos são mais baixos^8^. Novos marcadores mais específicos de formação e de reabsorção óssea têm sido estudados para estabelecer correlações com a histologia óssea, entretanto, apenas o PTH e a FA têm se mostrado úteis na prática clínica pediátrica^9^. Quanto à avaliação de exames de imagem em pediatria, até a presente data não há recomendações para realização de densitometria, tomografia de alta resolução ou ressonância. Da mesma forma, a realização de radiografia de abdome lateral para avaliação de calcificações vasculares ainda não está bem estabelecida para essa faixa etária^1,10,11^ Outros exames, como variação de onda de pulso, ultrassonografia com Doppler de carótidas e tomografia computadorizada com escore de cálcio, são indicados em casos específicos e em pesquisas^10^. Na prática clínica, habitualmente realiza-se a radiografia simples, para avaliação de idade óssea, fraturas, sinais de raquitismo, manifestações tardias do hiperparatireoidismo e de calcificações metastáticas^10^. A análise histológica do tecido ósseo obtido por meio de biópsia é o padrão ouro para diagnóstico da DMO-DRC. Recomenda-se a realização de biópsia óssea em crianças com DRC em casos de deformidade ou dor óssea grave, fratura atraumática, hipercalcemia ou hipofosfatemia persistentes, apesar da otimização do tratamento^10^.

A despeito da gravidade da DMO-DRC na faixa etária pediátrica, percebe-se que o diagnóstico é complexo. Registros internacionais que estudam crianças com DMO-DRC^12^ e também nacionais, com adultos^13,14^, destacam-se como ferramentas valiosas para a compreensão dessa problemática. No Brasil, não há estudo multicêntrico sobre a DMO-DRC pediátrica. A exemplo da contribuição desses registros, vislumbra-se a necessidade desse registro brasileiro sobre a DMO-DRC na faixa etária pediátrica.

Na fase inicial, o projeto DOMINÓ tem como objetivo geral descrever as características epidemiológicas, clínicas, laboratoriais, radiológicas e de biópsia óssea da DMO-DRC em pacientes pediátricos brasileiros. Os objetivos específicos incluem: (1) identificar a prevalência da DMO-DRC por meio do perfil bioquímico, (2) analisar a associação entre DMO-DRC e os dados clínicos, morbidade (fraturas, déficit de crescimento, deformidades e dor óssea) e mortalidade, (3) propor diretrizes de diagnóstico e tratamento da DMO-DRC pediátrica.

O estudo prospectivo observacional analisará desfechos como crescimento, morbidade cardiovascular, fraturas, resposta ao tratamento e mortalidade a partir de coletas de dados semestrais e avaliação anual, por um período de três anos, podendo se estender.

## Métodos

### Desenho do Estudo E População

O Registro **DOMINÓ** será um estudo de coorte retrospectivo e prospectivo, multicêntrico e observacional. A etapa inicial analisará os dados dos pacientes prevalentes em 2024, enquanto o estudo prospectivo analisará os dados a partir de 2025. Serão elegíveis os pacientes pediátricos com DRC estágios 3 a 5-D e transplantados renais, com idade até 17 anos, 11 meses e 29 dias, dos referidos centros.

A amostra será de conveniência, pois todas as crianças e adolescentes dos centros participantes, com os critérios de inclusão, serão convidados a participar. Estima-se uma amostra de 700 pacientes. A coleta será realizada pelos pesquisadores dos referidos centros, a partir de dados dos prontuários.

Serão incluídos pacientes cujos pais/responsáveis legais aceitarem participar e assinarem o termo de consentimento livre e esclarecido, e para os participantes maiores de 10 anos, também a assinatura do termo de assentimento livre e esclarecido.

### Variáveis de Análise

*Variáveis demográficas*: Nome do centro, número do paciente, iniciais do paciente, data de nascimento, sexo, raça/cor da pele, etiologia da DRC, data de início do acompanhamento, tipo de tratamento da DRC, data de início da 1ª diálise, modalidade dialítica, data de mudança de modalidade da diálise, data do transplante, data da avaliação atual. Esses dados estão detalhados na Tabela 1.

**Tabela 1:** Dados demográficos

Dados demográficos
Nome do centro Número do paciente Iniciais do paciente Data de nascimento Sexo (1. Feminino; 2. Masculino) Raça/cor da pele (1. Branco; 2. Não branco) Etiologia da DRC (1. Uropatia; 2. Glomerulopatia; 3. Doenças hereditárias; 4. Doenças císticas; 5. Outras) Data de diagnóstico da DRC/Início do acompanhamento Tipo de tratamento da DRC (1. Conservador; 2. Diálise; 3. Transplante) Data de início da 1ª diálise Modalidade dialítica (1. Peritoneal; 2. Hemodiálise; 3. Hemodiafiltração) Data de mudança de modalidade da diálise (se houver) Data do transplante Data da avaliação atual

*Dados antropométricos e clínicos de acometimento ósseo*: Peso, altura e índice de massa corpórea (IMC) de acordo com as classificações da Organização Mundial da Saúde. Dados como: dor óssea, fraturas, local da fratura, deformidade óssea, limitação de deambulação pelo comprometimento ósseo, tumor marrom e outros sinais e sintomas serão categorizados (sim/não) Tabela 2.

**Tabela 2:** Dados antropométricos, clínicos e de tratamento

Dados antropométricos
Peso Z-Escore peso/idade Altura (cm) Z-Escore estatura/idade Classificação da estatura (1. baixa estatura; 2. estatura adequada; 3. alta estatura) Índice de Massa Corpórea (IMC) Z-Escore de IMC Classificação do IMC (1. baixo peso; 2. peso adequado; 3. sobrepeso/obesidade)
Dados clínicos de acometimento ósseo
Dor óssea (1. Sim; 2. Não) Fraturas (1. Sim; 2. Não) Deformidade óssea (1. Sim; 2. Não) Local da fratura (1. Membro inferior; 2. Membro superior; 3. Coluna; 4. Quadril; 5. Outros) Sinais de osteopenia (1. Sim; 2. Não) Limitação de deambulação pelo comprometimento ósseo (1. Sim; 2. Não) Tumor marrom (1. Sim; 2. Não) Outros sintomas (1. Sim; 2. Não)
Dados do tratamento da DMO-DRC
Tipo de quelante de fósforo utilizado (1. Não utiliza; 2. Carbonato de cálcio; 3. Sevelamer; 4. Outros) Dose de carbonato de cálcio quelante (dose em mg/kg/dia) Dose de carbonato de cálcio suplemento (dose em mg/kg/dia) Dose do sevelamer (dose em mg/kg/dia) Uso de calcitriol (1. Sim; 2. Não) Dose de calcitriol (mcg/kg/semana) Uso de paricalcitol (1. Sim; 2. Não) Dose de paricalcitol (mg/kg/dia) Uso de cinacalcete (1. Sim; 2. Não) Dose de cinacalcete (mg/kg/dia) Bicarbonato de sódio (1. Sim; 2. Não) Dose de bicarbonato de sódio (mEq/kg/dia) Uso de corticoide (1. Sim; 2. Não)Concentração de cálcio no dialisato (mmol/L) (1. 1,25; 2. 1,5; 3. 1,75; 4. Outra)

*Dados relacionados ao tratamento DMO-DRC*: Tipo e dose do quelante de fósforo, de vitamina D ou análogos (colecalciferol, calcitriol, paricalcitol, outros), calcimimético, bicarbonato de sódio, corticosteroide. Concentração de cálcio no dialisato. Os tipos de medicação serão analisados como variáveis categóricas e a dose como variável contínua (dose por quilo de peso) Tabela 2.

*Exames laboratoriais*: Cálcio sérico (mg/dL), fósforo sérico (mg/dL), fosfatase alcalina (UI/L), gama glutamil transferase (γGT U/L), 25-hidroxivitamina D (ng/mL), 1,25-diidroxivitamina D (ng/mL), PTHi (pg/mL), bicarbonato sérico (mEq/L). Os valores de normalidade serão definidos e avaliados de acordo com os protocolos e diretrizes atuais^11^. Como as faixas de normalidade dos ensaios de fosfatase alcalina e PTHi podem não ser iguais entre os centros, padronizaremos os dados desses marcadores em relação ao limite superior do normal (LSN). Esses dados estão detalhados na Tabela 3.

**Tabela 3:** Dados dos exames laboratoriais e de imagem

Dados laboratoriais
eTFG (tratamento conservador e transplantados) (Fórmula de Schwartz) Creatinina (mg/dL) Dosagem de creatinina (1. Jaffé, 2. enzimático) Ureia (mg/dL) Cálcio (mg/dL) Fósforo (mg/dL) Bicarbonato (mEq/L) FA (UI/L) VR FA (mínimo) VRFA (máximo) FA (xLSN) PTH (pg/mL) VR PTH (mínimo) VR PTH (máximo) PTH (xLSN) 25-hidroxivitamina D (ng/mL) 1,25-diidroxivitamina D (ng/mL) Gama glutamil transferase (U/L)
Radiografia simples
Idade óssea em meses (0. Não tem; 1. Normal; 2. Atrasada; 3. Adiantada) Mão e punho (0/1/2/3) Tíbia (0/1/2/3) Pelve (0/1/2/3) Crânio (0/1/2/3) Abdome lateral (0/1/2/3) (0. Não realizou; 1. Normal; 2. Sinais de osteopenia; 3. Fratura) Calcificação de partes moles (1. Sim; 2. Não) Fratura em outro sítio (1. Sim; 2. Não)
Densitometria óssea
Fez densitometria óssea (1. Sim; 2. Não) BMD (L1-L4) (g/cm²) BMC (L1-L4) (g) Área (cm^2^) % banco de dados de referência (%) BMC/bone size (g/cm^3^)
Ecocardiograma
Ecocardiograma (1. Sim; 2. Não) Massa de VE (g) Índice de massa (g/m^2,7^) Disfunção diastólica (1. Sim; 2. Não) Fração de ejeção (%) Calcificação valvar (1. Sim; 2. Não)

Abreviaturas – FA: fosfatase alcalina; PTH: paratormônio; VR: valor de referência; xLSN: número de vezes o valor normal; BMC: do inglês, *bone mineral content*; BMD: do inglês, *bone mineral density.*

*Exames de imagem*: As radiografias para avaliação de idade óssea, osteopenia, deformidades e fraturas serão analisadas pelo radiologista de cada instituição e os dados, categorizados (sim/não). A densitometria óssea seguirá as recomendações recentes^9,15^. O ecocardiograma avaliará disfunção miocárdica e calcificação valvar (Tabela 3).

*Dados de biópsia óssea*: Quando realizada por indicação clínica, avaliaremos o diagnóstico histológico por meio da classificação TMV [Turnover (remodelação), Mineralização e Volume]^1^, além de outros parâmetros de histomorfometria, detalhados na tabela suplementar.

### Processamento e Análise dos Dados

As variáveis serão coletadas em formulário estruturado através do Excel^®^. Cada centro terá sua planilha, a qual deverá ter checagem dupla. Estamos em processo de aprovação de um registro para que a inserção dos dados se faça de forma eletrônica, com proteção, de modo privado na Sociedade Brasileira de Nefrologia, onde a inclusão será feita por médicos autorizados, cadastrados e devidamente autenticados.

Os dados contínuos e semicontínuos serão analisados pelo teste de Shapiro-Wilk para determinação da normalidade. Média e desvio-padrão, ou mediana primeiro e terceiro quartis e/ou amplitude, serão expressos conforme apropriado. As comparações e correlações serão realizadas por testes paramétricos e não paramétricos de acordo. Os dados categóricos serão analisados por meio do teste qui-quadrado com significância. Será considerado para todo estudo risco α ≤ 0,05 para erro Tipo I.

### Aspectos Éticos

O presente estudo foi elaborado seguindo as Diretrizes e Normas Regulamentadoras de Pesquisas Envolvendo Seres Humanos do Conselho Nacional de Saúde propostas pelas Resoluções 466/12 e 510/16, visando à preservação dos quatro referenciais da bioética: autonomia, não maleficência, beneficência e justiça. Além disso, atende aos requisitos da Declaração de Helsinque para pesquisa em seres humanos. Atualmente, temos cinco centros com aprovação pelos respectivos Comitês de Ética, com previsão para inclusão de outros centros no Brasil, condicionada à aprovação pelo correspondente comitê de ética em pesquisa.

## Discussão

Considerando a elevada morbimortalidade da DMO-DRC pediátrica, o diagnóstico dessa entidade é extremamente importante. A avaliação da saúde óssea e do acometimento cardiovascular são elementos-chave nos cuidados dessas crianças e desses adolescentes.

Como já mencionado, os estudos sobre DMO-DRC pediátrica em nosso país são escassos. A análise dos dados do registro DOMINÓ poderá revelar preditores de resposta ao tratamento da DMO-DRC.

Os desafios desse estudo podem incluir o número de tabelas para preenchimento e o desinteresse dos pais/cuidadores em assinar o termo de consentimento. Apesar dos obstáculos que possam advir, torna-se importante conhecer os dados epidemiológicos, clínicos e terapêuticos dessa enfermidade com o objetivo de melhorar a morbidade, a mortalidade e a qualidade de vida desses pacientes. O estudo DOMINÓ será um grande registro de dados de DMO-DRC pediátrica no Brasil e permitirá estudos futuros sobre esse grande problema da DRC em crianças e adolescentes.

## Material Suplementar

O seguinte material on-line está disponível para o presente artigo:

Tabela S1 - Dados de biópsia óssea.
